# Dataset for multimodal fake news detection and verification tasks

**DOI:** 10.1016/j.dib.2024.110440

**Published:** 2024-04-16

**Authors:** Alessandro Bondielli, Pietro Dell'Oglio, Alessandro Lenci, Francesco Marcelloni, Lucia Passaro

**Affiliations:** a Department of Computer Science, University of Pisa, Largo Bruno Pontecorvo, 3, 56127, Pisa, Italy; b Department of Information Engineering, University of Pisa, Largo Lucio Lazzarino, 1, 56122, Pisa, Italy; c Department of Philology, Literature and Linguistics, University of Pisa, Via S. Maria 36, 56127, Pisa, Italy

**Keywords:** Fake news, Multimodal data, Data collection and annotation, Machine learning, Natural language processing

## Abstract

The proliferation of online disinformation and fake news, particularly in the context of breaking news events, demands the development of effective detection mechanisms. While textual content remains the predominant medium for disseminating misleading information, the contribution of other modalities is increasingly emerging within online outlets and social media platforms. However, multimodal datasets, which incorporate diverse modalities such as texts and images, are not very common yet, especially in low-resource languages. This study addresses this gap by releasing a dataset tailored for multimodal fake news detection in the Italian language.

This dataset was originally employed in a shared task on the Italian language. The dataset is divided into two data subsets, each corresponding to a distinct sub-task. In sub-task 1, the goal is to assess the effectiveness of multimodal fake news detection systems. Sub-task 2 aims to delve into the interplay between text and images, specifically analyzing how these modalities mutually influence the interpretation of content when distinguishing between fake and real news. Both sub-tasks were managed as classification problems.

The dataset consists of social media posts and news articles. After collecting it, it was labeled via crowdsourcing. Annotators were provided with external knowledge about the topic of the news to be labeled, enhancing their ability to discriminate between fake and real news. The data subsets for sub-task 1 and sub-task 2 consist of 913 and 1350 items, respectively, encompassing newspaper articles and tweets.

Specifications Table

**Table:** 

Subject	Data Science
Specific subject area	Multimodal Fake News Detection and Cross-modal Relation Classification in Fake and Real News.
Data format	Rows in each .tsv file contain the link to the text and/or image, and the associated labels
Type of data	.tsv (dataset with links and labels)
Data collection	The dataset was collected from Twitter and then annotated via crowdsourcing. First, 920,054 tweets and 128,611 news articles were collected from Twitter using keywords about the Russo-Ukrainian war. The data span from Feb. to Dec. 2022. Then, we exploited a manually collected set of already verified fake news and misleading claims to gather from this data a number of news which were likely to be fake. Second, a human annotation process was performed through Prolific. For each sub-task, five annotators were provided with the verified fake news as context and asked to label a few items. Only those items for which at least three out of the five annotators provided the same label were kept in the dataset.
Data source location	Tweets and articles were collected from Twitter.
Data accessibility	Repository name: Mendeley Data Data identification number: 10.17632/s3mfjxcg68.1 Direct URL to data: https://data.mendeley.com/datasets/s3mfjxcg68/1
Related research article	Bondielli, A., Dell'Oglio, P., Lenci, A., Marcelloni, F., Passaro, L. C., Sabbatini, M. (2023). Multi-fake-detective at Evalita 2023: MULTI-Fake-DetectiVE at EVALITA 2023: Overview of the MULTImodal Fake News Detection and VErification Task. In Proceedings of the Eighth Evaluation Campaign of Natural Language Processing and Speech Tools for Italian. Final Workshop (EVALITA 2023), CEUR. org, Parma, Italy. https://ceur-ws.org/Vol-3473/paper32.pdf

## 1 Value of the Data

• The dataset represents a valuable resource for multimodal fake news detection, offering data through two main perspectives: standard fake news detection, enriched with the multimodal setting (sub-task 1), and the relation between text and image in propagating deceitful information (sub-task 2).
• Although researchers are becoming increasingly interested in the problem of fake news detection, the available resources (datasets) are few and often not multimodal.
• The dataset offers a valuable addition to the few available multi-modal resources for fake news detection.
• The dataset also offers a valuable addition to the resources in the Italian language for the fake news detection problem.
• The dataset can easily be used to train machine and deep learning classifiers, to test new Large Language Models (LLMs) and Vision-Language models (VLMs) under few-shot or zero-shot learning, or even to carry out analyses on data containing verified fake news.

## 2 Background

The MULTI-Fake-DetectiVE dataset has been exploited to analyze the impact of multimodality in the Fake News Detection problem [1] in the context of EVALITA 2023 [2].

In the last few years, the online dissemination of fake news has seen a substantial increase [3], particularly in the context of real-world events reported as breaking news. Entities with malicious intents tend to exploit breaking news to push their agenda by distorting facts and deliberately disseminating false or misleading information.

The distorted use of online social media platforms has become evident during the initial phase of the COVID-19 pandemic, which emerged in what has been defined by several authors as a Post-Truth Era [4], characterized by the dominance of emotions and pseudo-facts [3]. This phenomenon intensified with the outbreak of the Russian war against Ukraine.

The proliferation of these issues over the years led to the creation of several initiatives dedicated to fake news detection, and related tasks such as fact-checking. The topic has gained significant relevance in the research community. The corpus of literature on fake news detection and disinformation is constantly growing despite the inherent challenges and the multifaceted nature of the problem.

## 3 Data Description

The MULTI-Fake-DetectiVE (MULTImodal Fake News Detection and Verification) dataset played a pivotal role during the namesake shared task [1] organized in the context of the EVALITA 2023 evaluation campaign of Italian Natural Language Processing systems [2]. The shared task was focused on the exploration of multimodality in the context of fake news. The creation of a dataset of fake news which include both textual and visual components is of paramount importance for better analyzing the Fake News Detection Problem. Approaches and techniques proposed in the last few years mostly focus on the uni-modal (i.e., text only) setting [3,5]. Most of these approaches rely on either the actual content of the news (i.e., the text itself), its context (e.g., social network structures, temporal information), or a combination of both [6]. However, images can be utilized in the context of disinformation in several ways [3]. For example, an image could enhance the credibility of a piece of text containing fake news, or could be described in such a way that the original content is misinterpreted by the reader.

The MULTI-Fake-DetectiVE repository contains two sub-folders corresponding, respectively, to the two sub-tasks, namely the Multimodal Fake News Detection and the Cross-modal relation classification in Fake and Real news. Each of the two sub-folders contains the data pertaining to the corresponding sub-task, consisting in (i) a *training set*, which contains data collected from February 2022 to September 2022, (ii) a *contemporary test set*, which includes data collected in the same time window as the training set, and (iii) a *future test set*, which includes data collected in a subsequent time window, specifically from October 2022 to December 2022.

We describe below the training set, and the contemporary and future test sets for each sub-task.

### 3.1 Sub-task 1

The first sub-task, named Multimodal Fake News Detection, is aimed at modeling the fake news detection problem in a multiclass and multimodal perspective. The corresponding dataset is contained in the Multimodal-Fake-News-Detection sub-folder. The news are classified in the following four classes:

­ *Certainly Fake*: news that is certain to be fake, whatever the context. The corresponding label in the dataset is 0.
­ *Probably Fake*: news that is likely to be fake, but may include some real information or at the very least be somewhat credible. The corresponding label in the dataset is 1.
­ *Probably Real*: news that is very credible but still retains some degree of uncertainty about the provided information. The corresponding label in the dataset is 2.
­ *Certainly Real*: news that is certain to be real and incontestable, whatever the context. The corresponding label in the dataset is 3.

The Multimodal-Fake-News-Detection sub-folder contains three .tsv files, consisting of the training set, the contemporary test set and the future test set.

Tables 1 and 2 illustrate the structure of the dataset, providing examples of entries. The partitioning of the two tables is only for the sake of clarity in representation. Actually, both non-aggregated and majority votes are encompassed within the same .tsv files.

**Table 1:** Examples of entries with non-aggregated ratings in the dataset concerning the sub-task 1.

ID	Rating 1	Rating 2	Rating 3	Rating 4	Rating 5
3110807	3	1	1	1	2
3038029	2	2	0	2	2
1498003150414008322	0	2	2	2	1

**Table 2:** Examples of individual entries with aggregated rating in the dataset concerning the sub-task 1.

ID	URL	Type	Label
3110807	https://t.co/EdyIsGXRmY	Article	1
3038029	https://t.co/Xnf9Jw03WY	Article	2
1498003150414008322	https://twitter.com/anyuser/status/1498003150414008322	Tweet	2

Tables 1 and 2 refer to the same instances as examples, and they are connected through the ID column. In Table 1, the non-aggregated rating values are shown. Each label is represented by a numerical value ranging from 0 to 3, corresponding to a specific class (0 for Certainly Fake, 1 for Probably Fake, 2 for Probably Real, and 3 for Certainly Real). These represent the annotation provided by individual annotators during the crowdsourcing annotation task, as detailed in Section “EXPERIMENTAL DESIGN, MATERIALS AND METHODS”.

In Table 2, each row includes the ID, the URL of the tweet (or the news article), the Type of the data (if it is a tweet or an article), and the Label. The latter corresponds to the label which received at least three votes among the five annotators. In case of uncertainty, that is, when less than 3 out of the 5 annotators provided the same label, we removed the instance.

**Fig. 1:**
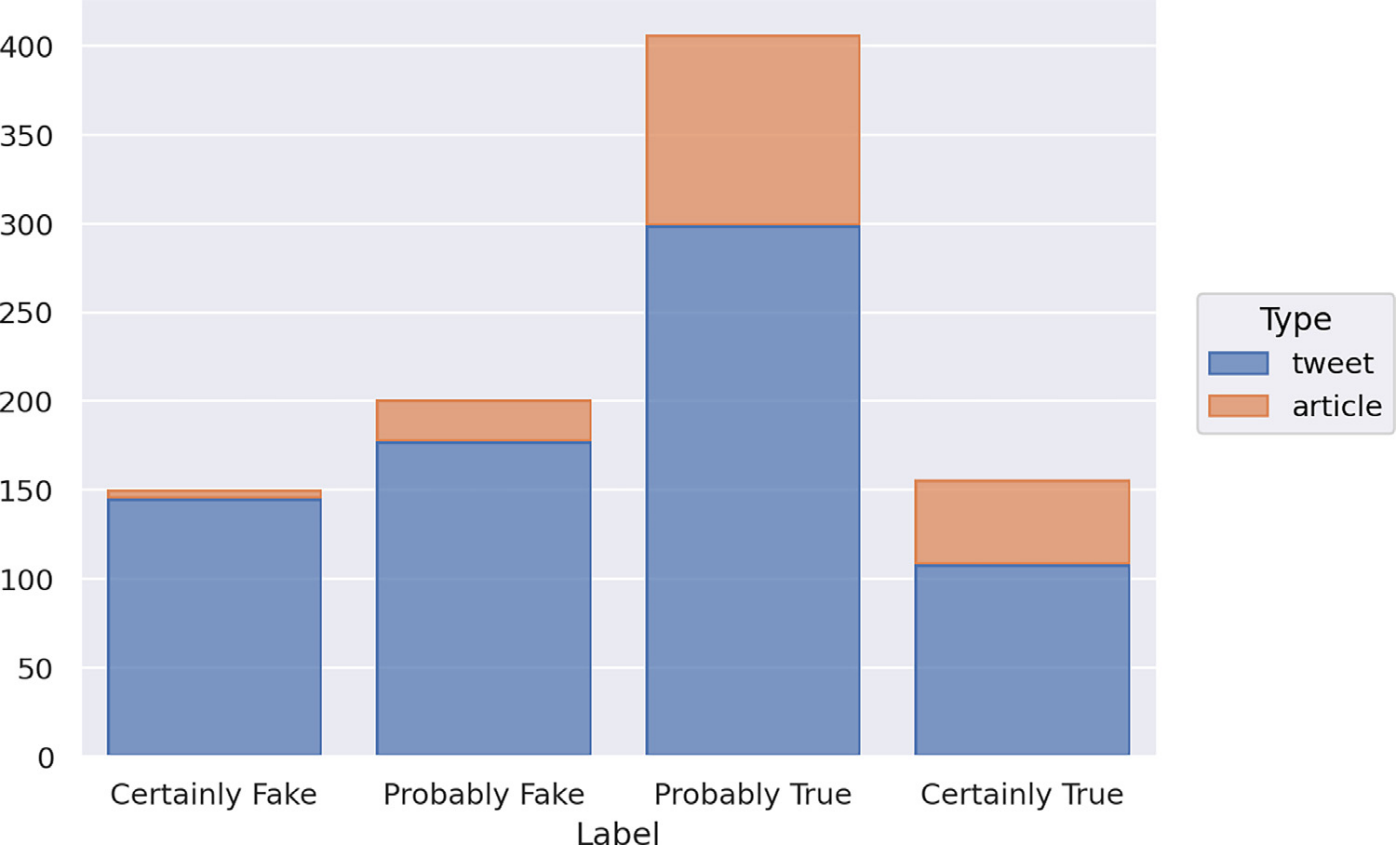
Distribution of the news of the training set in the four classes and with respect to the type of data.

The training set includes 913 instances, specifically 729 tweets and 184 articles. Fig. 1 illustrates the distribution of the training set instances in the four classes and with respect to the types of data (article or tweet).

Regarding the distribution of the items of the training set among the classes, there are 150 items labeled as Certainly Fake, 201 as Probably Fake, 406 as Probably Real and 156 as Certainly Real, with, in total, 351 items belonging to the fake classes, and 562 belonging to the real classes. Both types of data, tweets and articles, have a similar distribution of fake and non-fake instances, with a higher number of instances in real classes than in the fake ones. In the case of articles, 5 instances are labeled as Certainly Fake, 24 as Probably Fake, 107 as Probably Real and 48 as Certainly Real. In the case of tweets, 145 items are labeled as Certainly Fake, 177 as Probably Fake, 299 as Probably Real and 108 as Certainly Real.

**Fig. 2:**
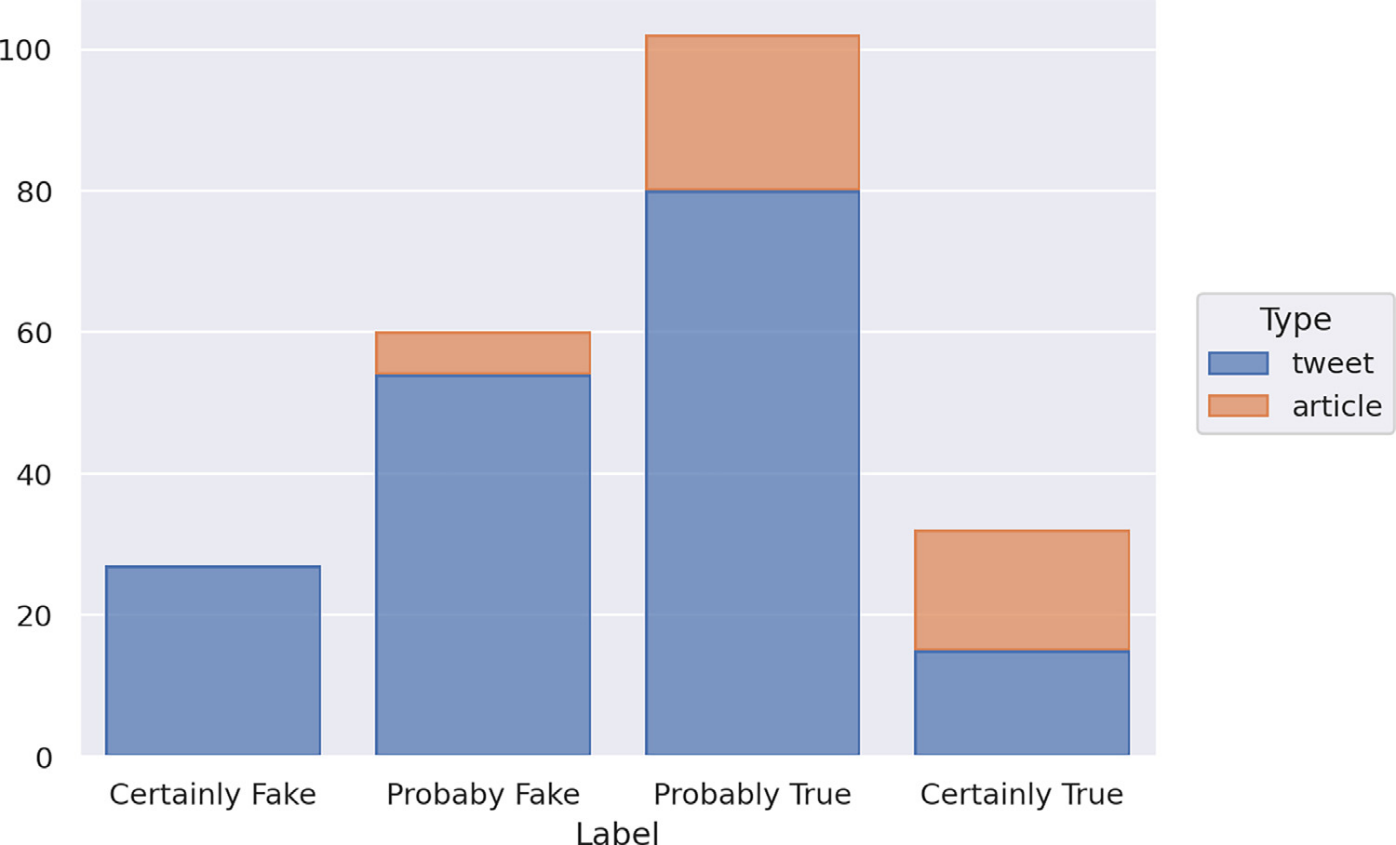
Distribution of the news of the contemporary test set in the four classes and with respect to the type of data.

The contemporary test set includes 221 items from the same time window as the training set. It contains 45 articles and 176 tweets. Fig. 2 illustrates the distribution of the contemporary test set items in the classes and with respect to the types of data (article or tweet).

The contemporary test set includes 27 items labeled as Certainly Fake, 60 as Probably Fake, 102 as Probably True, and 32 as Certainly True. The set of the articles consists of 0 Certainly Fake, 6 Probably Fake, 22 Probably True, and 17 Certainly True instances and the set of tweets contains 27 Certainly Fake, 54 Probably Fake, 80 Probably True, and 15 Certainly True instances.

**Fig. 3:**
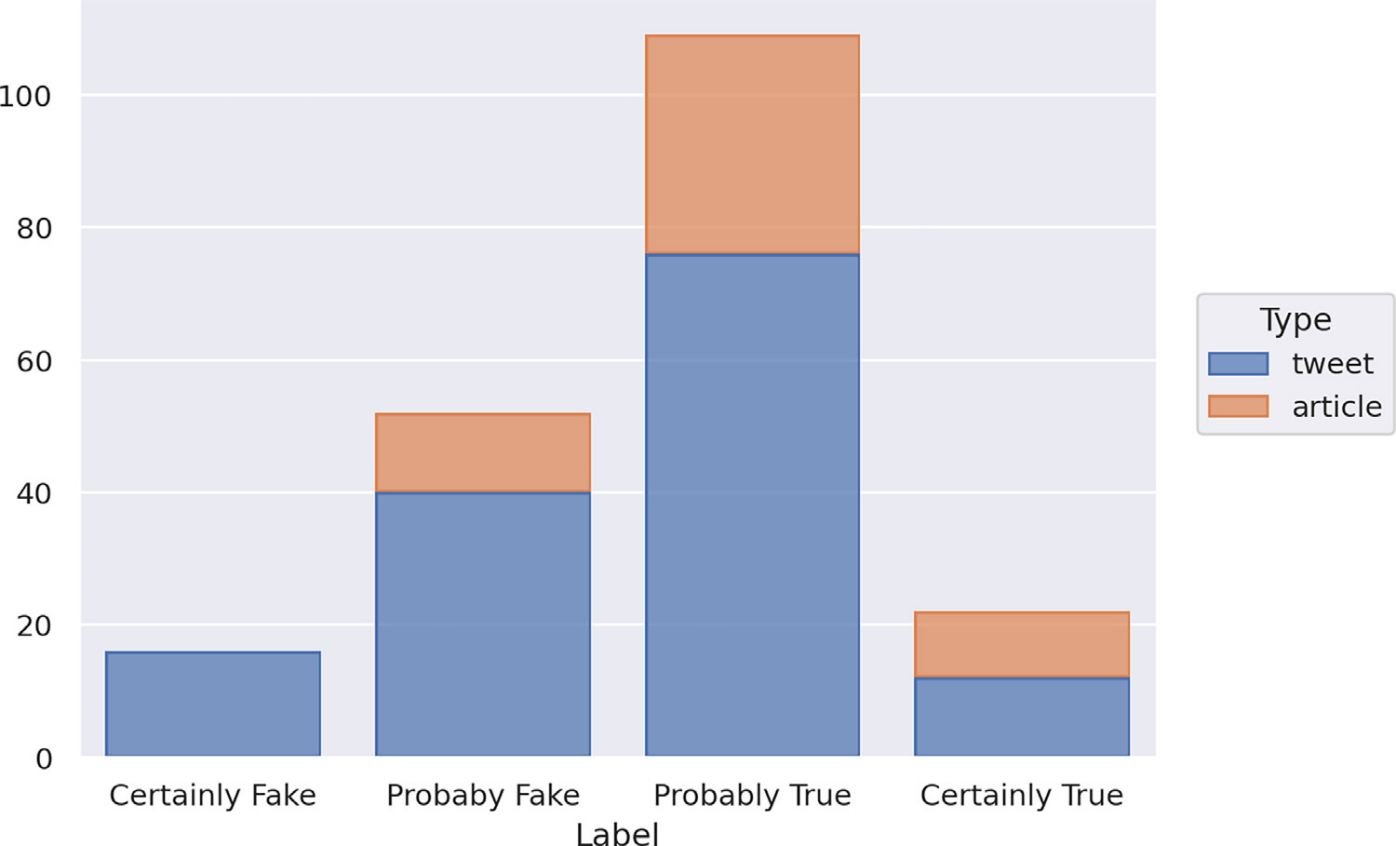
Distribution of the news of the future test set in the four classes and with respect to the type of data.

The future test set includes 199 items collected from October 2022 to December 2022, a subsequent timespan to the one of the training set. This set was built to assess possible differences in performance in case of potential concept drift, that is, when the distribution of the data changes along the time. Thus, we might have differences between data from the same distribution as the one of the training set and data from a possibly different distribution subsequent to the one of the training set. It includes 55 articles and 144 tweets. Fig. 3 shows the distribution of the future test set instances in the four classes and with respect to the type of data (article or tweet).

The future test set includes 16 instances labeled as Certainly Fake, 52 as Probably Fake, 109 as Probably True, and 22 as Certainly True. The set of the articles contains 0 Certainly Fake, 12 Probably Fake, 33 Probably True, and 10 Certainly True instances, and the set of tweets contains 16 Certainly Fake, 40 Probably Fake, 76 Probably True, and 12 Certainly True instances.

Table 3 summarizes the distribution of instances in sub-task 1.

**Table 3:** Distribution of the items in the dataset for sub-task 1.

	Certainly Fake	Probably Fake	Probably Real	Certainly Real
Training set	150	201	406	156
Test (Contemporary) set	27	60	102	32
Test (Future)	16	52	109	22
Total	193	313	617	210

### 3.2 Sub-task 2

The dataset for the second sub–task, referred to as Cross-modal Relation Classification in Fake and Real News, is contained in the Cross-modal-Relation-Classification-in-Fake-News sub-folder. This dataset is designed to investigate the interaction between the textual and the visual modalities in the context of fake and real news. The primary objective is to provide insights on how images and texts in fake and real news may contribute to misleading interpretations of the content related to the other modality and to the news as a whole. The problem is modeled as a three-class classification task, and the classes are defined as follows:

­ *Misleading*: either the textual or the visual component is used deceptively to induce the misinterpretation of the other. The corresponding label in the dataset is 0.
­ *Not Misleading*: the combination of the visual and textual components does NOT induce the misinterpretation of the news. The corresponding label in the dataset is 1.
­ *Unrelated*: the visual component is not related to the text component or does not add information to the text component or does not change its interpretation in a significant way. The corresponding label in the dataset is 2.

The Cross-modal-Relation-Classification-in-Fake-News sub-folder contains three .tsv files, corresponding to the training set, the contemporary test set and the future test set, respectively.

Tables 4 and 5 illustrate the structure of the dataset, providing examples of entries. As for sub-task 1, the partitioning of the two tables is only for the sake of clarity in representation.

**Table 4:** Examples of entries with non-aggregated ratings in the dataset concerning the sub-task 2.

ID	Rating 1	Rating 2	Rating 3	Rating 4	Rating 5
1500046876963393536	2	2	2	2	2
3033998	1	1	2	2	1
3029515	2	2	1	0	2

**Table 5:** Examples of individual entries with aggregated rating in the dataset concerning the sub-task 2.

ID	URL	Type	Label
1500046876963393536	https://twitter.com/anyuser/status/1500046876963393536	Tweet	2
3033998	https://t.co/C6u8vcw5sP	Article	1
3029515	https://t.co/9KudkFq90Z	Article	2

Tables 4 and 5 present the same instances linked through the ID columns. Table 4 shows the non-aggregated ratings, while Table 5 provides the Label entries along with other relevant details.

**Fig. 4:**
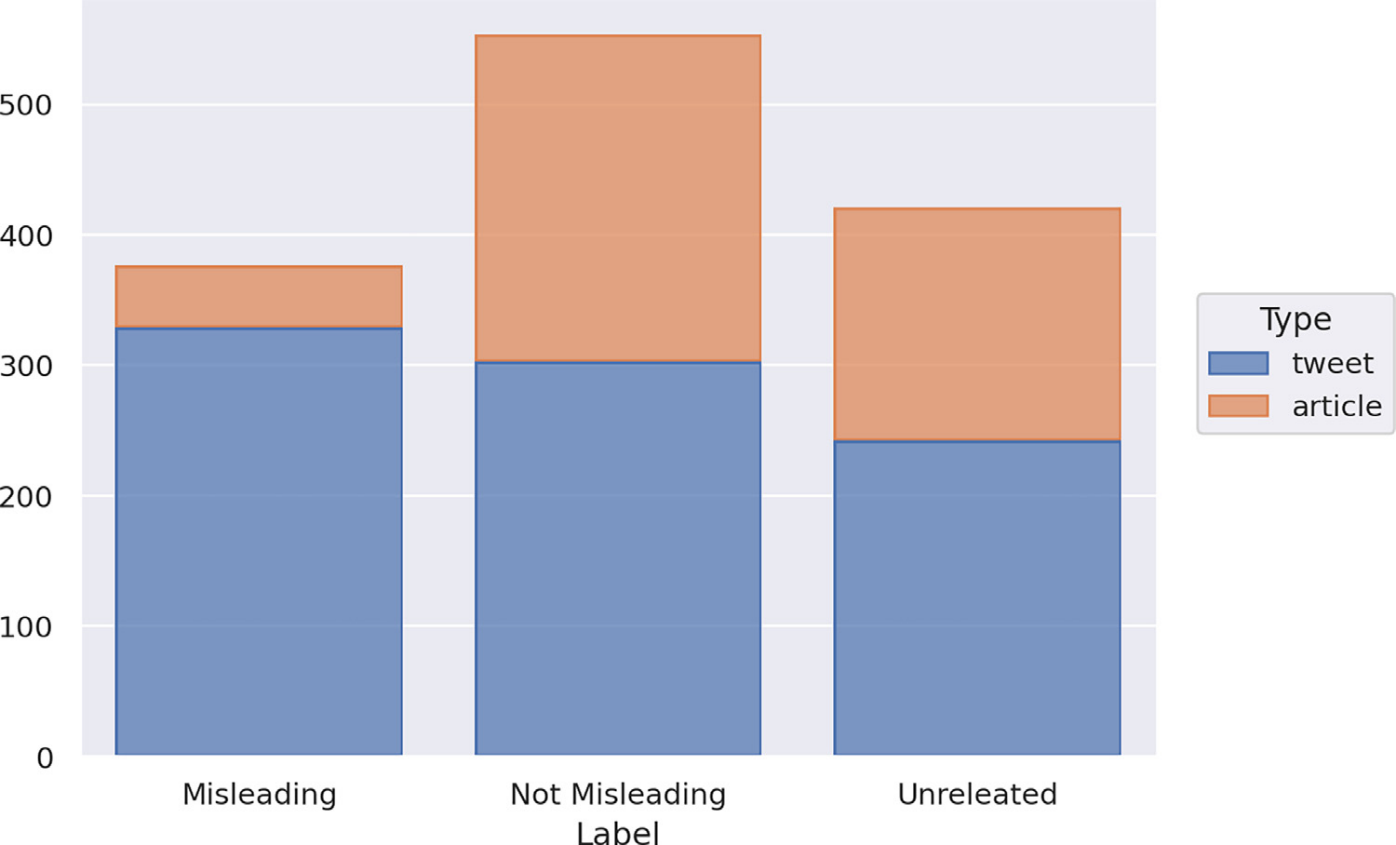
Distribution of the news of the training set in the three classes and with respect to the type of data.

The training set contains a total of 1,350 instances, distributed into 874 tweets and 476 articles. In the training set, there are 376 instances labeled as Misleading, 553 as Not Misleading, and 421 as Unrelated. Fig. 4 shows the distribution of the news of the training set in the three classes and with respect to the type of data (article or tweet).

The three classes are quite balanced in the whole dataset, with a slight majority of the *Not Misleading* class. In the case of the articles, the distribution is unbalanced with respect to the *Misleading* class. Indeed, 47 articles are labeled as Misleading, 250 as Not Misleading, 179 as Unrelated, while in the case of tweets, 329 instances are labeled as Misleading, 303 as Not Misleading, 242 as Unrelated. There are far fewer articles than tweets, and a disparity emerges on the number of articles and tweets labeled as Misleading, while the other two classes are balanced also with respect to the type of data.

**Fig. 5:**
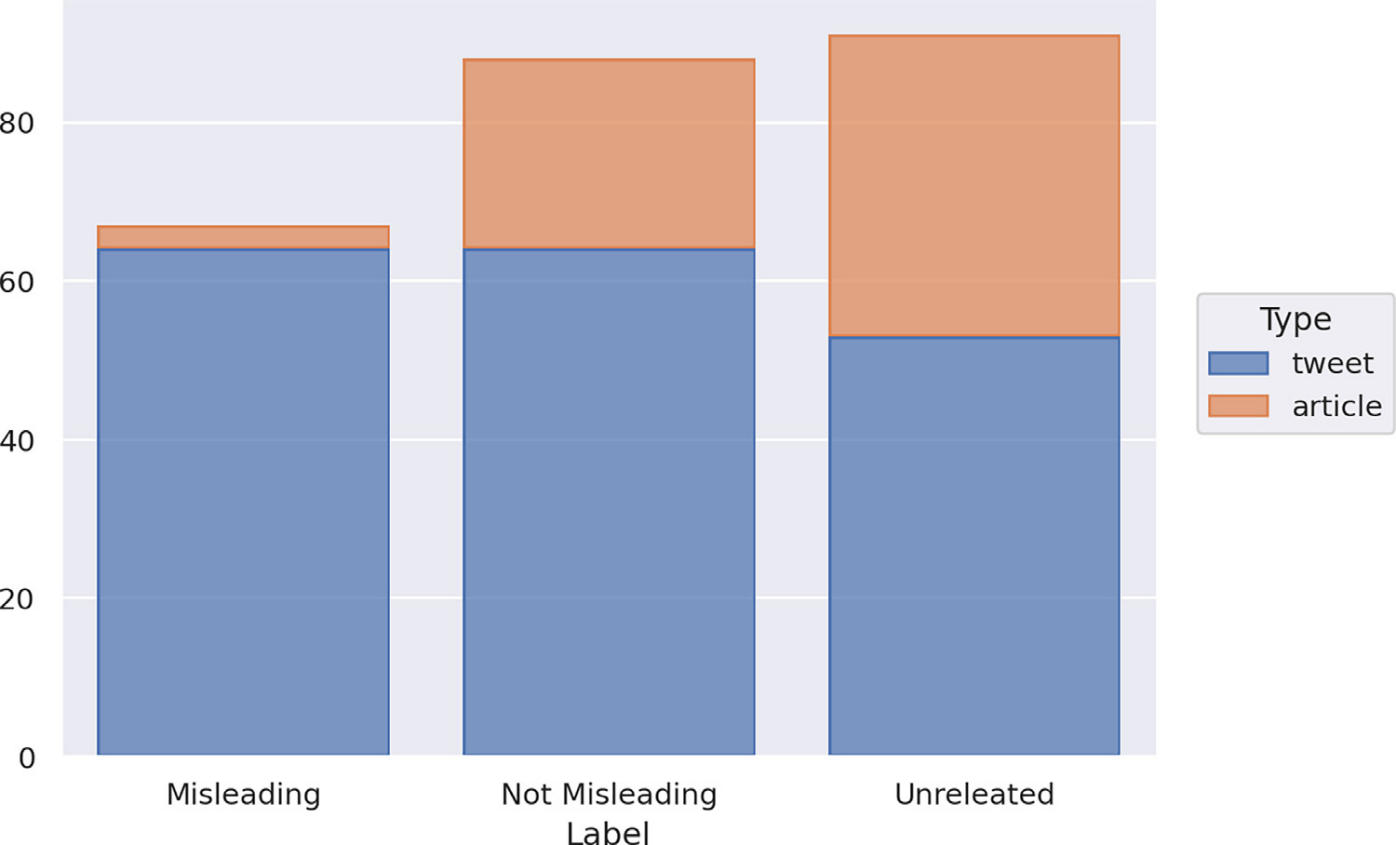
Distribution of the news of the contemporary test set in the three classes and with respect to the type of data.

The contemporary test set contains 246 items, with 65 articles and 181 tweets. Similar to sub-task 1, Fig. 5 shows the distribution of the news of the contemporary test set in the three classes and with respect to the type of data (article or tweet).

The contemporary test set contains 67 items labeled as Misleading, 88 as Not Misleading, 91 as Unrelated. In particular, it contains 3 Misleading, 24 Not Misleading and 38 Unrelated articles, and 64 Misleading, 64 Not Misleading and 53 Unrelated tweets.

**Fig. 6:**
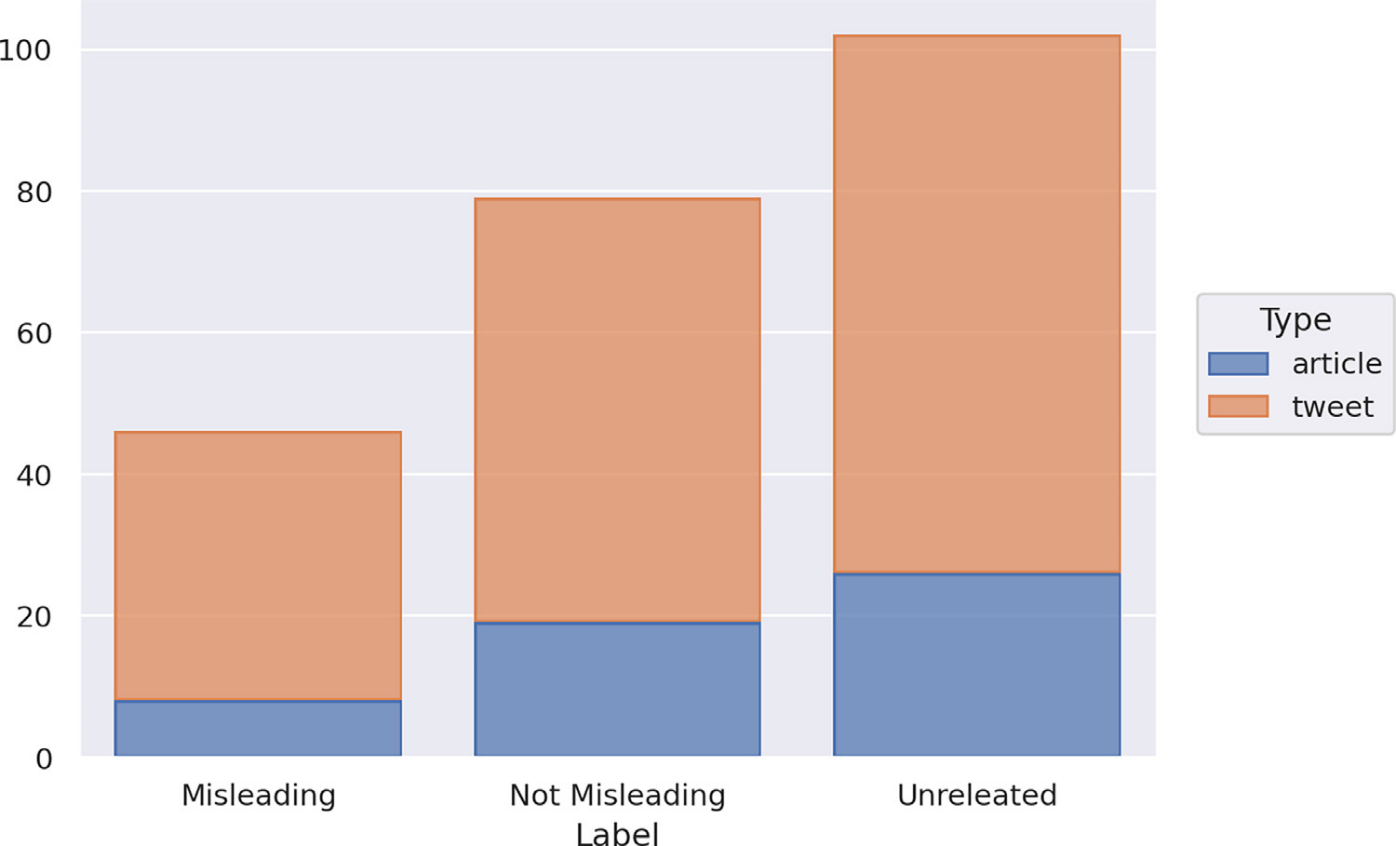
Distribution of the news of the future test set in the three classes and with respect to the type of data.

The future test set includes 227 items, with 53 articles and 174 tweets. Fig. 6 shows the distribution of the future test set news in the three classes. The test set contains 46 items labeled as Misleading, 79 as Not Misleading, 102 as Unrelated. In particular, it contains 8 Misleading, 19 Not Misleading and 26 Unrelated articles, and 38 Misleading, 60 Not Misleading and 76 Unrelated tweets.

Table 6 summarizes the distribution of items in sub-task 2.

**Table 6:** Distribution of the items in the dataset for sub-task 2.

	Misleading	Not Misleading	Unrelated
Training set	376	553	421
Test (Contemporary) set	67	88	91
Test (Future)	46	79	102
Total	472	677	619

Table 7 summarizes the total number of items for each sub-task with respect to the training set, the contemporary test set, and the future test set. It also shows the total distribution of the images obtainable from the URLs of each set, and the distribution of the images with respect to the type of data (if it is tweet or article). The values refer to those obtained at the time of dataset creation. It is worth noting that the number of instances is not aligned with the number of images: a single instance may be related to more than one image.

**Table 7:** Distribution of items and images in the dataset.

	Sub-task 1			Sub-task 2		
	Training	Contemporary test	Future test	Training	Contemporary test	Future test
Articles	184	45	55	476	65	53
Tweets	729	176	194	874	181	174
Datapoints	913	211	199	1350	246	227
Articles Images	1481	706	978	476	1404	438
Tweets Images	873	217	196	1038	225	231
All Images	2354	923	1174	1514	1629	669
Avg. Images Per Article	8.04	15.68	17.78	1.00	21.60	8.26
Avg. Images Per Tweet	1.19	1.23	1.01	1.18	1.24	1.32

## 4 Experimental Design, Materials and Methods

**Fig. 7:**
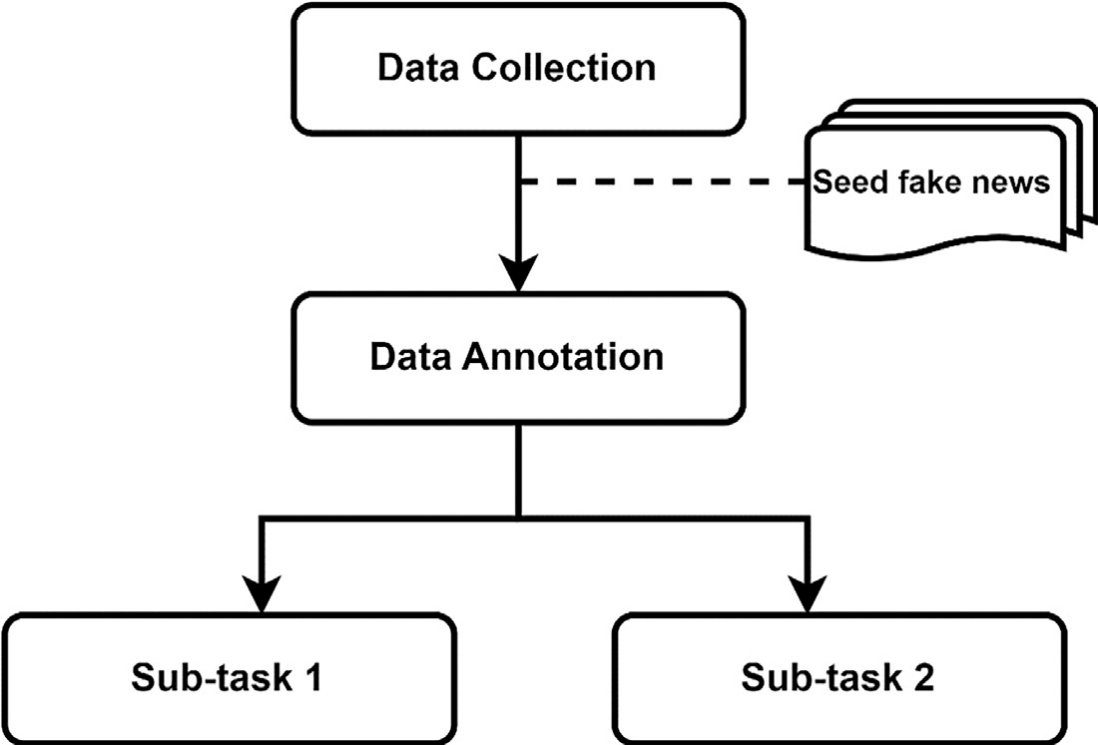
Flowchart of the multi-step process.

The dataset was collected and annotated through crowdsourcing, following a multi-step process inspired by the one proposed in [7]. As shown in the flowchart in Fig. 7, the process consists of two main steps, namely data collection and data annotation. We report below the details of each step.

### 4.1 Data collection

We first gathered Twitter data related to the Ukrainian-Russian war in a selected time span (from February 2022 to December 2022). To collect such data, we identified a set of keywords (in Italian) that were representative of the conflict. The keywords used are: *ucraino, Ucraina, ucraina, ucraini, ucraine, Russia, russo, russi, soldato, soldati, civile, civili, guerra, Putin, Zelensky, NATO, Chernobyl, Bucha, Kharkiv, Azov*.

Then, we collected texts and images from tweets. In particular, we identified news articles linked in tweets, and we downloaded their source text and images. Note that for the MULTI-Fake-DetectiVE task, we gave participants only the “primary image” (i.e., the cover image in the website) of each article, and not all images. For tweets, we collected the text and all the images posted along with the tweet. Notably, during this phase, data were collected without specific consideration for the sub-tasks.

Finally, we utilized a manually collected set of verified fake news and misleading claims, referred to as “seed fake news and misleading claims”, to generate the dataset for each sub-task. During this process, we considered various news outlets reporting on the fake news as well as fact-checking websites. These seed fake news and misleading claims served a dual purpose.

On the one side, they were employed to filter the original dataset by assessing their semantic similarity with data samples. In addition to this, we randomly selected other items regardless of their similarity with them. This step aimed to ensure two essential aspects:

­ The resulting dataset would have included only pertinent elements directly related to the Ukrainian-Russian war.
­ The class distribution for both sub-tasks would not have been excessively skewed in favor of real news and non-misleading claims, as it might occur in an uncontrolled scenario.

On the other side, the seed fake news and misleading claims served as context for the data annotation process described below.

### 4.2 Data annotation

For the data annotation process, we performed a crowdsourcing task with Prolific^1^ to acquire labels for our sub-task datasets.

For each sub-task, we provided annotators with the seed fake news and misleading claims as context. This external knowledge about the topic of the news has been shown to enhance the annotators’ ability to discriminate between fake and real news [7].

**Fig. 8:**
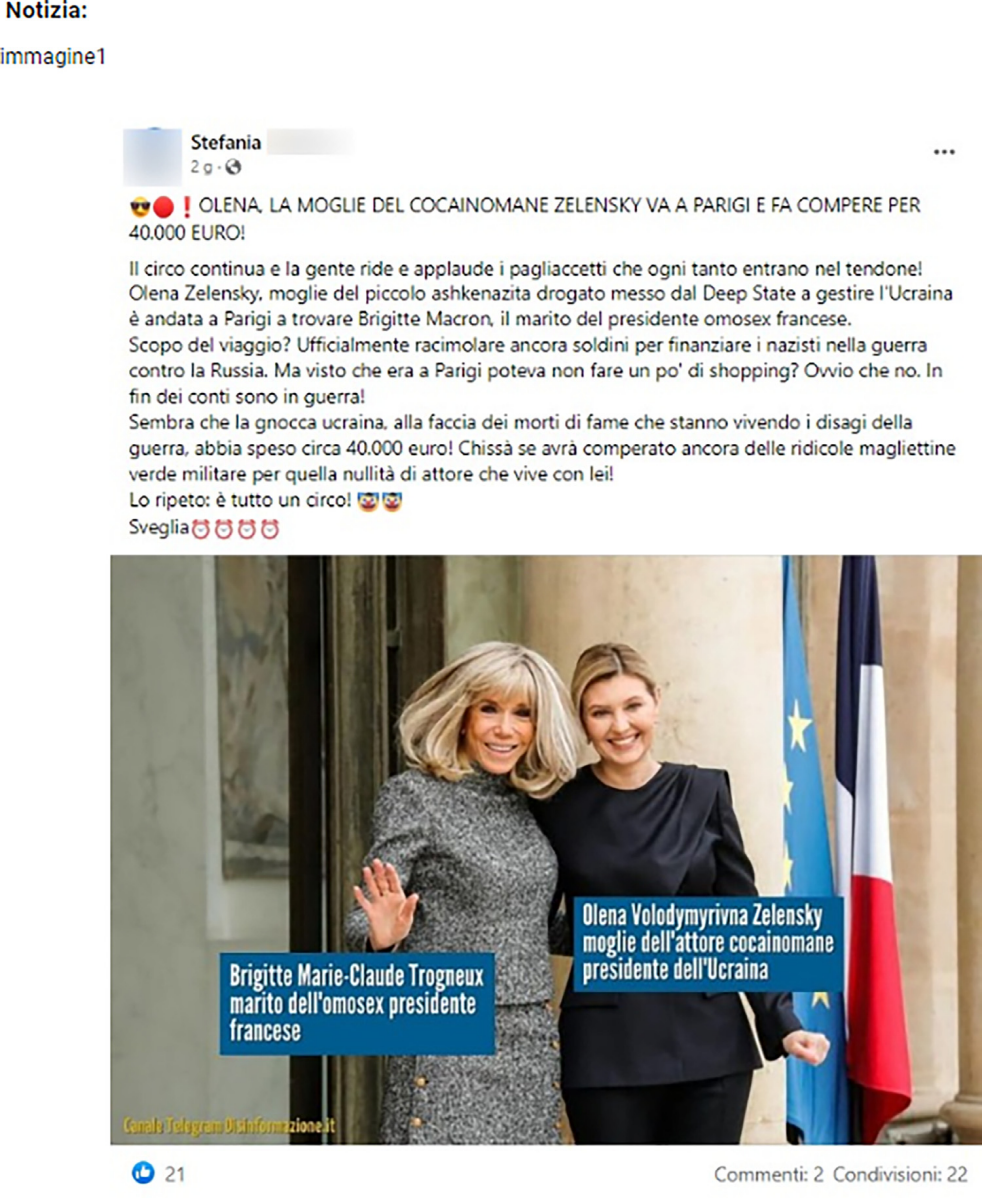
An example of “Certainly Fake” news for the sub-task 1. *Source*: Open (https://www.open.online/2022/12/22/foto-olena-zelenska-shopping-parigi-40000-fake-fc/).

**Fig. 9:**
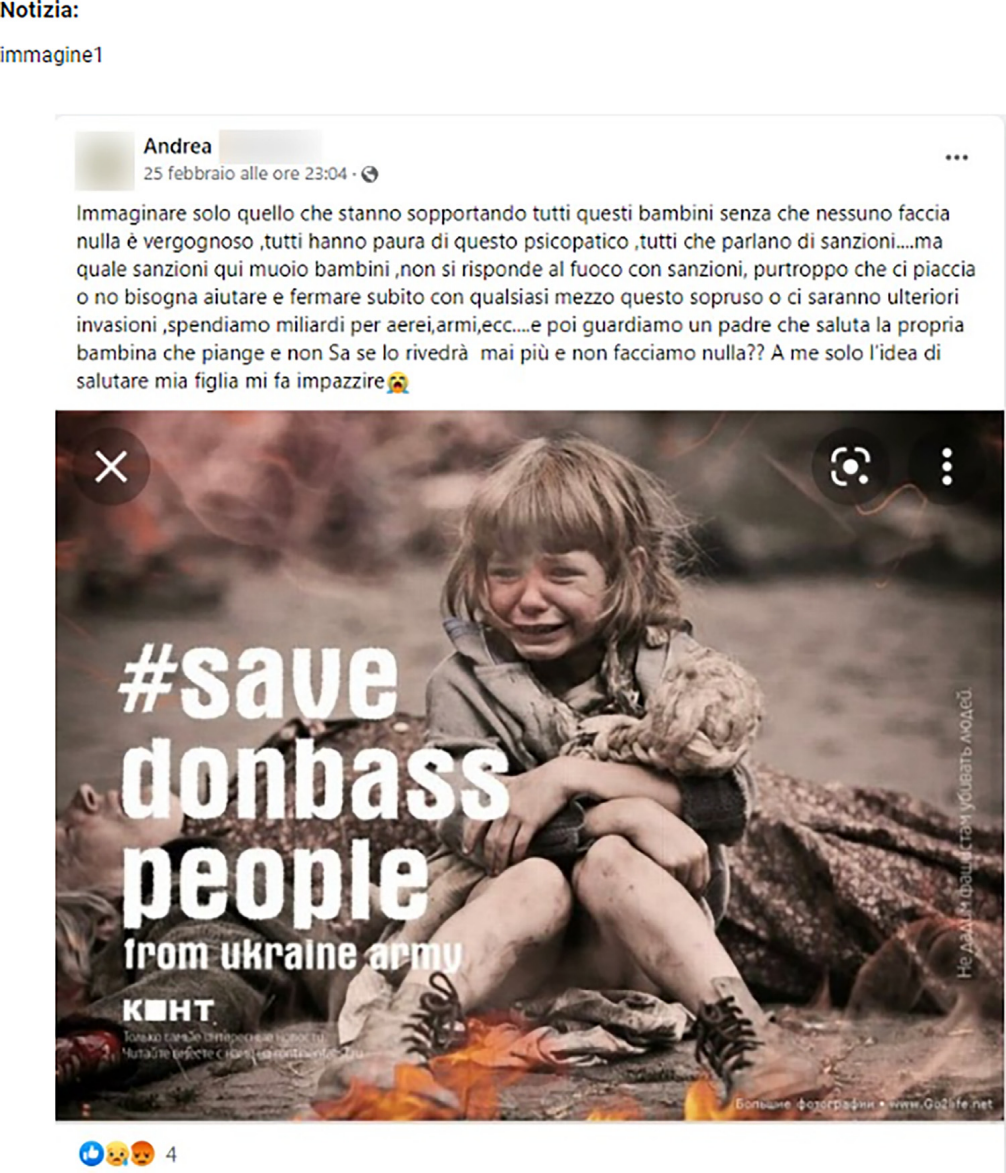
An example of “Misleading” pair of news and image for the sub-task 2. *Source*: Open (https://www.open.online/2022/03/04/foto-bambina-vittima-donbass-1941/).

To regulate the duration of the annotation experiments and maintain the annotators' focus during the annotation task, the dataset was segmented into distinct batches. Each batch, comprising approximately 30 items, was evaluated by a panel of five distinct annotators. It is important to highlight that, as a consequence of this annotation process, the ratings presented in Tables 1 and 4 do not exclusively correspond to individual subjects. In other words, the values in the “Rating 1” column, for instance, are not all generated by a single annotator. Before starting with the annotation task, the subjects were presented with different information, including the instructions, the list of seed fake news and misleading claims, and a list of examples for each of the labels. A news labeled “Certainly Fake” provided to the annotators as an example for sub-task 1 is presented in Fig. 8. Fig. 9 shows an example of Misleading content (text and image) for sub-task 2.

The post in Fig. 8 is Certainly Fake because in the image it is stated that Brigitte Macron is a man (it is a fake news that has been circulating for some time) while Olena Zelensky, wife of Volodymyr Zelensky, is described as the wife of a cocaine addict, another dated hoax. Furthermore, Olena was accused of having spent 40.000 euros during her visit to Paris, even if there is no evidence.

The picture in Fig. 9 is misleading with respect to the news. Indeed, the picture was taken during the filming of a movie about the Nazi invasion of the Soviet Union in 1941. Thus, the girl in the image is not a victim of Donbass, as it is stated in the text, but was simply used in a deceptive way by pro-Russian propaganda.

## Inter-Annotator Agreement

In sub-task 1, inter-annotator agreement was computed as the average Spearman correlation coefficient between annotator pairs, considering the ordered nature of the labels. The average correlation was 0.43 (σ= 0.04).

For sub-task 2, Fleiss' Kappa was utilized to measure the inter-annotator agreement, given that the labels were not inherently ordered. The obtained value was k= 0.25.

## Limitations

Datasets refer to Twitter data. The guidelines and accessibility of the instances included in them are subject to changes over time.

## Ethics Statement

The data was collected respecting the Twitter guidelines at the time of collection. The annotations were collected through a crowdsourcing task on the Prolific platform, following their guidelines for fair compensation. Informed consent was obtained from participants of the task. Data and annotations have been fully anonymized*.*

## CRediT authorship contribution statement

**Alessandro Bondielli:** Conceptualization, Methodology, Software, Data curation, Writing – original draft, Writing – review & editing, Visualization, Investigation, Supervision. **Pietro Dell'Oglio:** Conceptualization, Methodology, Software, Data curation, Writing – original draft, Visualization, Investigation, Validation. **Alessandro Lenci:** Conceptualization, Methodology, Software, Writing – review & editing. **Francesco Marcelloni:** Supervision, Writing – review & editing. **Lucia Passaro:** Conceptualization, Methodology, Software, Data curation, Writing – original draft, Writing – review & editing, Visualization, Investigation.

## Data Availability

Multi-Fake-DetectiVE (Original data) (Mendeley Data).

## Footnotes

1 https://www.prolific.com/.
